# Immunological Analysis of Isothiocyanate-Modified α-Lactalbumin Using High-Performance Thin Layer Chromatography

**DOI:** 10.3390/molecules26071842

**Published:** 2021-03-25

**Authors:** Jenny Spöttel, Johannes Brockelt, Svenja Badekow, Sascha Rohn

**Affiliations:** 1 Institute of Food Chemistry, Hamburg School of Food Science, University of Hamburg, Grindelallee 117, 20146 Hamburg, Germany; jenny.spoettel@chemie.uni-hamburg.de (J.S.); johannes.brockelt@web.de (J.B.); svenja.badekow@studium.uni-hamburg.de (S.B.); 2 Department of Food Chemistry and Analysis, Institute of Food Technology and Food Chemistry, Technische Universität Berlin, TIB 4/3-1, Gustav-Meyer-Allee 25, 13355 Berlin, Germany

**Keywords:** whey proteins, allergenicity, α-lactalbumin, isothiocyanates, protein modifications, protein antigenicity, peptide antigenicity, HPTLC immunostaining, food processing

## Abstract

Undirected modifications between food proteins and secondary plant metabolites can occur during food processing. The results of covalent interactions can alter the functional and biological properties of the proteins. The present work studied the extent of which covalent conjugation of the bioactive metabolite benzyl isothiocyanate (BITC; a glucosinolate breakdown product) to the whey protein α-lactalbumin affects the protein’s allergenicity. Additional to the immunological analysis of native untreated and BITC-modified α-lactalbumin, the analysis of antigenic properties of proteolytically digested protein derivatives was also performed by high performance thin layer chromatography and immunostaining. As a result of the chemical modifications, structural changes in the protein molecule affected the allergenic properties. In this process, epitopes are destroyed or inactivated, but at the same time, buried epitopes can be exposed or newly formed, so that the net effect was an increase in allergenicity, in this case. Results from the tryptic hydrolysis suggest that BITC conjugation sterically hindered the cleavage sites for the enzyme, resulting in reduced digestibility and allergenicity. Residual antigenicity can be still present as short peptide fragments that provide epitopes. The desire to make food safer for allergy sufferers and to protect sensitized individuals from an allergenic reaction makes it clear that the detection of food antigens is mandatory; especially by considering protein interactions.

## 1. Introduction

Interactions between proteins and secondary plant metabolites occur frequently in nature, and can also arise during food production and processing. The effects of covalent protein modifications are diverse and can influence protein folding and structure as well as various biological (hydrolysis, antigenic and antimicrobial activity), and technofunctional and functional properties such as viscosity, gelation, foaming, solubility, emulsification, color, odor, and taste [1]. From a physiological point of view, a conjugation of secondary plant metabolites or their degradation products with dietary proteins may reduce the availability of the health-promoting secondary metabolites or decrease the bioavailability of essential amino acids [2]. However, covalent addition of natural, hydrophobic, electrophilic plant compounds is also considered a promising method to specifically affect protein functionality [3,4,5,6]. In this respect, a bunch of studies have already studied the functional and biological properties of proteins by their modification with secondary plant metabolites. For example, it was reported that a covalent interaction of selected polyphenols with the whey protein β-lactoglobulin (β-LG) altered its functional properties and reduced its allergenic activity [7]. Rade-Kukic et al. (2011) concluded that binding of isothiocyanates to β-LG changed the protein’s folding and structure, improving its technofunctional properties such as heat aggregation, foam formation, and emulsification [3]. Almost a handful of studies confirmed the change in the secondary and tertiary structure of proteins as a result of isothiocyanate (ITC) conjugation [6,8,9,10,11]. ITC are degradation products of glucosinolates, which are mainly found in *Brassicales* plants such as broccoli, cauliflower, brussels sprouts, and cabbage and are associated with various health-promoting properties (e.g., antibacterial, anti-inflammatory, and antidiabetogenic activity) [2,4,6,12,13,14,15,16]. Due to their functional group, isothiocyanates possess a high electrophilicity at the carbon atom, which makes a reaction with nucleophiles conceivable. The electrophilic center of ITC reacts with thiol and amino groups in the side chains of proteins to form thiocarbamates and thioureas [17,18,19]. Keppler et al. (2017) showed that covalent conjugation of allyl isothiocyanate (being a degradation of the glucosinolate sinigrin) to a whey protein isolate significantly affected the physicochemical properties such as charge, aggregation, surface hydrophobicity, and secondary structural features of the protein, depending on pH value [6]. It was also shown that a modification of these proteins with allyl isothiocyanate had no significant effect on the antibacterial activity of this protein against different strains of *Staphyolococcus aureus* and *Eschericha coli* [6].

The reason for an intensified research on whey proteins is because of their very high nutritional quality, being due to their high content of branched, sulfur-containing, and essential amino acids in an advantageous composition. Besides their high biological value, whey proteins are characterized by their extraordinary functional properties and their solubility over a wide pH value range, making them valuable food ingredients [3,20,21,22,23]. Whey proteins include various albumins and globulins, among which α-lactalbumin (α-LA) is the second most common protein in cow’s milk, accounting for 2–5% [20]. α-LA is a small, acidic protein, consisting of 123 amino acids and a molecular weight of 14.2 kDa. In addition, it is an important Ca^2+^-binding model protein and a classic example of the molten globule state. It is a component of lactose synthase [20,24]. For example, α-LA and its hydrolysates have been found to have an antihypertensive effect in hypertension [25], to contribute to stress reduction [26], and to regulate cell growth [27]. It further provides antimicrobial [28] and immunostimulatory properties [29]. Besides to the positive properties of α-LA, it is one of the main allergens in cow’s milk, along with β-LG and casein [30].

In general, food allergies are mostly type 1 (immediate-type) allergies that are mediated by specific IgE antibodies bound on mast cells. Binding of the antibody to the antigen activates the mast cells and stimulates them to degranulate. There is a release of histamine and a number of other mediators of allergic inflammation [31]. Type I allergenicity of the immediate reaction is a particular immunogenicity. While immunogenicity describes the ability to induce a humoral or cellular immune response, antigenicity describes the ability to be specifically recognized by antibodies produced as a result of an immune response to the given substance or molecule [32]. A substance that is recognized by the organism as an antigen is capable of eliciting an immune response and therefore has an immunogenic potential, the extent of which depends, among other things, on the molecular size and chemical structure. In the literature it is described that immunogenic substances usually must have a molecular weight higher than 5000 Daltons and contain antigenic regions in the secondary and tertiary structure, so-called epitopes. Generally, various proteins in food can therefore have an allergenic potential, because the epitopes can be specifically recognized by antibodies and subsequently trigger an allergic reaction. The extent of immunogenicity depends on the abundance and density of the epitopes. These epitope structures of proteins can be formed as linear (sequential and continuous) or conformational (discontinuous) epitopes [33,34,35,36,37]. The latter are formed by the folding of spatial structures such as the secondary or tertiary conformational arrangements, while sequential epitopes are short sections of the primary structure of proteins [31,38].

Despite cow’s milk being a valuable food source for humans and especially for infants, these food allergens can cause allergic reactions in sensitive people. However, there is a growing desire to make food safer for allergic sufferers and to further investigate the problem of milk allergy. As food allergens are a widespread health problem, many investigations have been made to modify milk ingredients to reduce or eliminate their allergenic potential [39,40,41]. It is well known that immune-influencing properties are characterized by immunogenicity and antigenicity, being related to protein structures. Therefore, it can be assumed that modifications of proteins influence the immune properties of the native protein. In the past, the influence of various food processing such as lactic acid fermentation, glycation, heat treatment, hydrostatic pressure and enzymatic hydrolysis was studied [42]. The results showed that proteins can aggregate, denature, or bind to lipid structures by the aforementioned technologies. They can also be glycosylated or glycated [43]. Obviously, these processing-induced structural and chemical alterations of the proteins are accompanied by a change in immunogenicity and allergenicity [43,44]. A large part of the current state of knowledge about the influence of the food processing on protein’s structure and function is based on numerous studies of model foods, in particular whey proteins from cow’s milk [45]. Not all food processing has the potential to reduce milk allergenicity. Increasingly, it is reported that allergenicity may be decreased, increased, or even remain unchanged by food techniques [46]. Although knowledge in this field is constantly improving, strategies to control milk allergy have not yet been satisfactorily solved. As the processing of milk proteins can affect the protein’s structure and function in various ways and thus, its allergenicity, this topic should be an important focus of future research considering a change of the allergenicity of milk proteins. In addition, there is a need for a robust and thoroughly evaluated and validated method for food allergenicity risk assessment that considers both protein digestion and protein analysis [47]. In fact, many studies focused on the investigation of the covalent interaction between whey proteins and isothiocyanates and its effect on the structural, functional, and selected nutritional properties of the proteins [3,4,6,8,9,10,11], while the studies regarding the influence on allergenic properties are insufficient [1].

However, analysis of undirected protein modifications and their implications for allergenicity is quite challenging. In contrast to other separation techniques, high performance thin layer chromatography (HPTLC) offers several advantages to study the influence of undirected posttranslational modifications that significantly affect the polarity, solubility, and respective properties of the protein. With little effort, it is possible to use different solvent systems, allowing a wide polarity range to be covered. This is supported by the variety of available chromatographic stationary phases. By varying the mobile and stationary phases, HPTLC can respond quite easily and quickly to the separation problem at hand. Biller et al. (2015) succeeded in developing a concept for the development of solvent systems, enabling intact proteins to be analyzed by HPTLC and providing a basis for the development of specific detections [48]. As the potential BITC-protein adducts described above can heavily influence the separation behavior, HPTLC separation might be advantageous. Even when not separated and remaining at the starting point, it is possible to recognize the behavior of the (modified) proteins. In a high-performance liquid chromatography (HPLC) approach, such compounds are discriminated, as they get stuck at the beginning of the analytical column or already in the pre-column. Another advantage of HPTLC compared to other separation techniques is the variety of compatible detection methods. For example, a chromatogram can be evaluated with different staining reagents, with coupling to mass spectrometry, or with a bioactivity-driven and effect-directed analysis [49,50,51]. The combined strategy of HPTLC followed by effect-directed detection via the specific antigen-antibody reaction, known as HPTLC immunostaining (HPTLC-IS) [52], can be used to analyze the biological function of allergenicity of proteins. The direct application of the bioassay to the analytical plate eliminates the time-consuming transfer of analytes, such as in immunoblotting, thereby further increasing the applicability of the method. Several studies already described antibody-based detection methods coupled to HPTLC: Meisen et al. (2011) succeeded in detecting glycosphingolipids [53], while Morschheuser et al. (2016) presented the detection of phosphorylated peptides using antibodies [54]. Another study by Morschheuser et al. (2017) dealt with the immunological investigation of proteins in milk after chromatographic separation [52].

**Figure 1:**
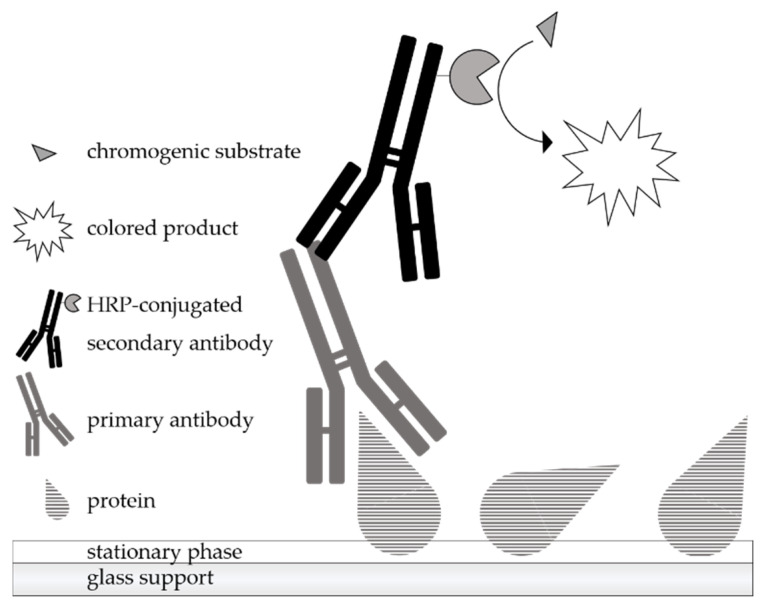
Schematic representation of the principle of an indirect antibody-based detection of proteins after chromatographic separation (HPTLC-IS). The primary antibody having affinity for the target protein is associated with the HRP-conjugated secondary antibody specific for the host of the primary antibody. Visualization of the ligated antibodies is achieved by the formation of a colored precipitate derived from a chromogenic substrate.

A scheme of the principle of immunostaining is shown in Figure 1. After the chromatographic separation of the samples, the analytes are detected by a protein antigenicity assay directly on the HPTLC plate. The immunological staining procedure used is very similar to the indirect enzyme immunoassay. The post-chromatographic detection starts with a blocking step to avoid non-specific binding of the detection antibodies on the surface of the separation material. Afterwards, the incubation is performed with a primary antibody that has an affinity for the target protein. The primary antibody used in the present work was a polyclonal anti-α-LA antibody from the organism rabbit. The secondary antibody needs to be specific for the host animal of the primary antibody and is conjugated with an enzyme, here, horseradish peroxidase (HRP). For detection, an enzymatic conversion of the chromogenic substrate tetramethylbenzidine (TMB) into a colored precipitate is finally performed, making the first antibody visible.

The aim of the present work was to study adducts of the whey protein α-LA and a bioactive metabolite from *Brassicaceae* vegetables, exemplarily benzyl isothiocyanate (BITC), to subsequently estimate and compare the antigenicity of the BITC-treated and untreated proteins. In addition to the analysis of protein derivatives, the study of residual antigenicity of peptides after enzymatic hydrolysis, which may still carry epitopes, should not be underestimated and was also be investigated in the present work. For this purpose, a combined strategy of HPTLC and immunostaining was applied.

## 2. Results

### 2.1. HPTLC-Immunostaining (HPTLC-IS) of Intact Proteins and Its Validation

Primarily, following the work of Morschheuser et al. [52], the HPTLC method coupled with antibody-based detection was applied to the whey protein α-LA and subsequently validated.

Initially, the linear correlation of the method used was confirmed by analyzing the standard solutions in a concentration range between 0.2 µg and 3 µg α-LA. The linearity was verified by means of Mandel’s fitting test. These results obtained are comparable to the linearity ranges of other HPTLC applications. For example, the HPTLC method of the antimalarial substance artemisinin showed linearity in the concentration range of 0.03–0.120 µg [55]. Reim et al. (2015) presented an HPTLC analysis of pea saponins that showed a linear correlation in the range of 1.25–6.25 µg [56]. For proteins, Morschheuser et al. (2016, 2017) reported a linear correlation for the range of 0.1–1 µg lysozyme using aptamers as detecting agents [57] and a linearity between 0.075–2 µg β-LG using an antibody-based detection [52].

Additionally, in the present study, the LOD and LOQ were also determined based on DIN standards using the calibration method (Table 1). Compared to other semi-quantitative HPTLC analyses, the results obtained were comparable. For example, Reim et al. (2015) calculated an LOD of 0.6 µg ± 17% and an LOQ of 2.1 µg ± 5% [56]. Morschheuser et al. (2016) showed a LOD and LOQ of 0.063 µg ± 19% and 0.112 µg ± 19%, when using HPTLC-aptastaining (HPTLC-AS) [57]. When using HPTLC-IS, they determined an LOD of 0.062 µg ± 24% and an LOQ of 0.093 µg ± 22% [52]. The precision of the HPTLC-IS method was within the accepted range of 15% according to the FDA guidelines.

### 2.2. HPTLC-Immunostaining of BITC-Modified and Non-Modified α-Lactalbumin

**Figure 2:**
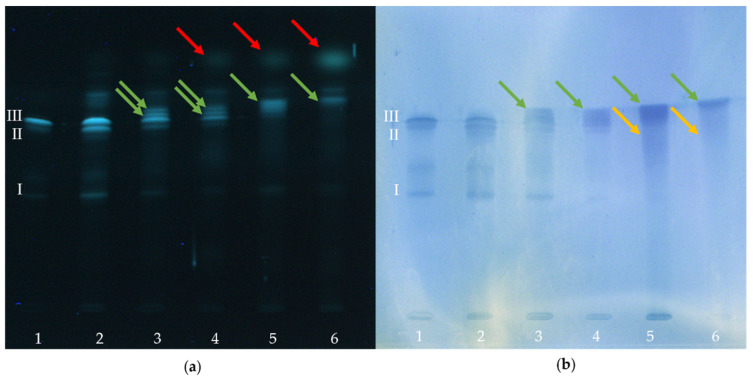
HPTLC analysis of BITC-α-LA derivatives as a function of the concentration of BITC. (**a**) Protein-specific staining with fluorescamine (UV-light; λ = 366 nm); (**b**) Immunostaining with antibodies (white light) for the exclusive detection of the antigen. (1) α-LA control (freshly prepared), (2) α-LA control (treated similar as BITC-protein derivatives), (3) BITC-α-LA derivative “low” (c_BITC_ = 3.8 mM), (4) BITC-α-LA derivative “moderate” (c_BITC_ = 38 mM), (5) BITC-α-LA derivative “high” (c_BITC_ = 75 mM); (6) BITC-α-LA derivative “very high” (c_BITC_ = 113 mM).

In the following, the influence of a conjugation between α-LA and BITC on the allergenic properties is presented. For this purpose, treated and untreated proteins were detected after chromatographic separation using the specific antigen-antibody reaction. Figure 2 shows a “twin plate” (one plate cut into two similar plates after the chromatographic separation) stained with the protein-specific dye fluorescamine (Figure 2a), detecting all proteins present. Figure 2b shows the chromatogram obtained from the immunological staining protocol.

In Figure 2, an α-LA solution freshly prepared in water was applied at position 1 of the plate. Lane 2 shows the α-LA control treated like the modifications, but without the addition of BITC. This control was intended to represent the effect of synthesis and re-conditioning of the protein. Lanes 3-6 show the protein derivatives “low”, “moderate”, “high”, and “very high” produced with increasing amounts of BITC.

The control samples applied as lanes 1 and 2 showed three bands (I, II, and III) for both staining procedures. When comparing the band sharpness between both detection methods, it was obvious that the band sharpness of HPTLC-IS slightly decreased. While the bands of the BITC-α-LA derivatives “low” and “moderate” were clearly separated from each other when stained with fluorescein (Figure 2a, lanes 3 and 4), overlapping and broadening of the bands already occurred in these samples during immunostaining (Figure 2b, lanes 3 and 4). This could be attributed to diffusion effects during the incubation in aqueous solutions [52].

When using a low concentration of BITC for producing the BITC-α-LA derivative “low”, both staining methods (Figure 2a,b; lane 3) showed bands that were assigned to non-modified, residual protein (I, II, III) as well as new additional bands (green arrows) with higher R_f_ values. For this protein derivative, both detection methods still showed a high agreement with the band pattern of the control sample (Figure 2a,b; lane 2).

With increasing concentrations of BITC, a steady decrease in intensity of the bands of the control (I, II and III) was observed, until they could not be detected anymore (Figure 2a,b; lanes 4–6). Similarly, a continuous increase in the intensity of new bands (green arrows) was observed with increasing BITC concentration (Figure 2a,b; lanes 4–6). The new bands had higher R_f_ values and appeared more intense and partially broadened. The trend obvious from Figure 2a supported the assumption that the ITC-protein adducts formed depending on BITC concentration to a certain extent.

In both figures, the formation of smeary streaks was pronounced at higher BITC concentrations (lanes 5 and 6, yellow arrows), which is probably due to an increase in the degree of modification. It is likely that there were numerous different protein modifications formed that impaired the chromatographic separation. Moreover, after the protein-specific staining with fluorescamine, another band can be detected attributable to an excess of residual BITC (red arrows). This identification was made by comparison with a standard BITC solution (comparison not shown here).

### 2.3. HPTLC-Immunostaining of Tryptic Peptides

**Figure 3:**
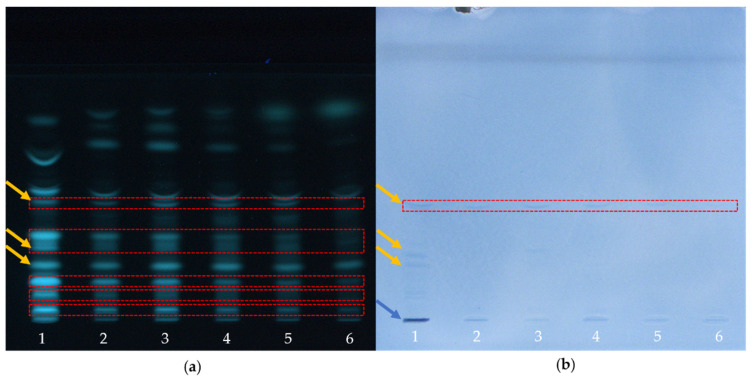
HPTLC analysis of tryptically digested α-LA derivatives as a function of BITC concentration. (**a**) Protein staining using fluorescamine as a derivation reagent (UV-light; λ = 366 nm), (**b**) Immunological staining (white light). (1) α-LA control (freshly prepared), (2) α-LA control (treated similar as BITC-protein derivatives), (3) BITC-α-LA derivative “low” (c_BITC_ = 3.8 mM), (4) BITC-α-LA derivative “moderate” (c_BITC_ = 38 mM), (5) BITC-α-LA derivative “high” (c_BITC_ = 75 mM); (6) BITC-α-LA derivative “very high” (c_BITC_ = 113 mM).

The present study showed that untreated and BITC-modified α-LA can be analyzed with regard to its antigenic properties using HPTLC-IS. In addition to the native protein structure, fragments/parts of the protein structure may also retain their antigenicity after proteolytic hydrolysis, so that antigenic analysis of corresponding peptides must not be neglected. Due to the low molecular weight of the peptides, their analysis by traditional methods such as Western blotting after gel electrophoresis is limited. Consequently, an immunological detection after HPTLC separation of the peptides resulting from the tryptic digestion of α-LA was also performed in the present work. For this purpose, α-LA was derivatized with BITC and subsequently hydrolyzed with the protease trypsin. Immunological detection by HPTLC-IS enabled the identification of the presence of residual epitopes in the hydrolysate. Figure 3 shows the HPTLC chromatograms of tryptic peptides after peptide/protein staining with fluorescamine (Figure 3a) and after immunostaining (Figure 3b). Antigenic and non-antigenic peptides can be differentiated.

Figure 3a shows a high agreement in the band pattern of both control samples (lanes 1 and 2). The number of bands detected was consistent. Only a slight, but uniform reduction in the intensity of all bands in lane 2 was observed. By comparing the band patterns of the control sample (lane 2) with those of the BITC-protein derivatives (lanes 3–6), a steady decrease in intensity was observed with increasing BITC concentration. Exemplarily, some bands, whose intensity decreased with increasing BITC concentration have been highlighted by red dashed boxes. In the case of an excess of BITC, some bands showed such a strong loss of intensity that some of them could no longer be detected (lanes 5 and 6; red dashed boxes). In Figure 3b, peptides with residual epitopes appear as distinct blue bands on a lighter background (yellow arrows). Based on this chromatogram, conclusions can be drawn about the residual antigenicity of the peptides. After enzymatic hydrolysis of the native α-LA using trypsin as protease, three blue bands were detected in the immunological assay (Figure 3b, lane 1; yellow arrows). The immunological staining showed a reduction in the number and a decrease in intensity of the blue band (red dashed box) with increasing modification with BITC (Figure 3b). Furthermore, a significant antigen-antibody reaction was observed for the initial application position of the sample (Figure 3b, lane 1, blue arrow). The intense blue coloration could be attributed to residual non-hydrolyzed and non-separated α-LA.

## 3. Discussion

**Figure 4:**
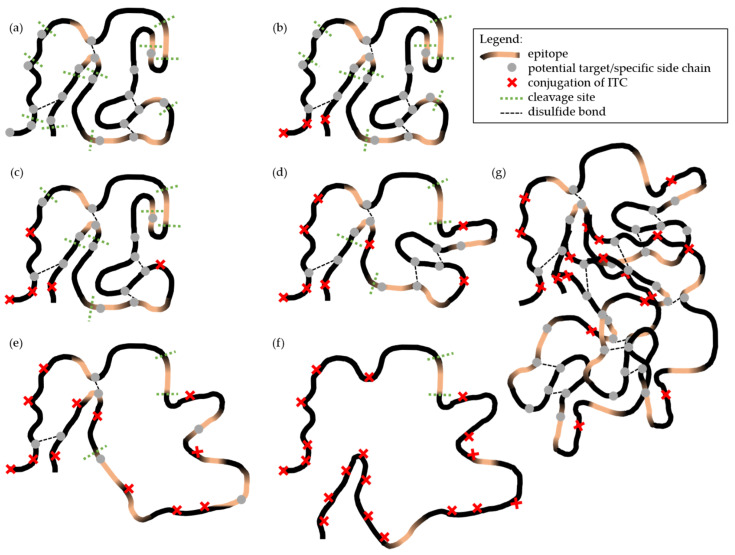
Schematic illustration of the influence of a protein modification on protein structure, epitopes, and enzymatic hydrolysis. While potential reaction sites for the ITC are represented by gray dots, ITC conjugation is highlighted by the red crosses. Intact epitopes are shown as orange regions, tryptic cleavage sites by green dashed lines, and disulfide bridges by black dashed lines. (**a**) Native protein with the potential reaction sites for ITC and its intact epitopes; (**b**–**g**) increasing degree of protein modification and the resulting change in protein structure, epitope, and enzymatic hydrolysis. The protein structure, locations of epitopes, disulfide bridges, and tryptic cleavage sites are not realistically depicted.

In the following, the previously described results from Figure 2 and Figure 3 will be illustrated and explained at hand of Figure 4. This figure serves to facilitate understanding and schematically depicts the influence of ITC conjugation on the structure, on the epitopes, and on the potential tryptic cleavage sites of the protein, when being successively modified with ITC, and depending on the degree of α-LA modification. It should be noted that only different model scenarios are illustrated, and that the discussion is based on a hypothetical approach for describing the impact of protein modifications on its antigenicity (creation and destruction of epitopes) in accordance with the current literature. Protein structure, the locations of epitopes, disulfide bridges, and tryptic cleavage sites are not depicted realistically, but only schematically for illustrating the different outcomes from a certain degree of modification possible.

In Figure 4a, the native protein is shown with potential reaction sites suitable for a reaction with ITC (gray dots) and with intact epitopes (orange regions). Here, no distinction is made between linear (sequential and continuous) or conformational (discontinuous) epitopes. The green dashed lines show the naturally occurring tryptic cleavage sites (trypsin cleaves exclusively C-terminal to arginine and lysine residues [58]), and the black dashed lines show the disulfide bridges in the protein [59]. In the native protein, the epitopes are intact and accessible to the antibody reaction [33,60,61,62,63], as reflected by the three blue bands in Figure 2b (lane 1, bands I, II, III). The high concordance of band patterns between control samples suggested that the synthesis and re-conditioning had no effect on the protein and its allergenicity (Figure 2a,b; lanes 1 and 2). The three bands detected in the native protein solution in lane 1 can be attributed either to the purity of the protein (≥85%) or to the three genetic variants of α-LA. It is noteworthy that the protein has two predominant variants A and B, and a third variant has been described, but was not yet approved [64].

The potential reaction sites, shown as gray dots in the figure, are replaced by red crosses in the case of a covalent bond between the nucleophilic groups of α-LA and the BITC. Previous studies confirmed the conjugation of ITC with the whey protein β-LG, determined by a decreased content of free amino and thiol groups in the protein, suggesting that ITC bind covalently to these protein side chains [3].

Using a low concentration of BITC for the preparation of the protein conjugates, a high agreement in the band pattern compared to the control sample (Figure 2a,b, lanes 2 and 3) can be seen in both detection techniques. It can be assumed that the use of a low BITC concentration resulted in a small number of protein modifications having no significant influence on the protein and its allergenicity. Despite the protein modifications with BITC, the epitopes appear to remain immunologically-active (Figure 2b, lane 3). This situation can be illustrated as in Figure 4b. It is likely that when using a low BITC concentration, only comparatively more easily accessible reaction sites in the outer part or the surfaces of the native protein structure were conjugated primarily, not noticeable influencing the whole protein structure with its epitopes.

For both detection techniques, two differences in the band pattern are highlighted with increasing concentration of BITC in Figure 2. On the one hand, with increasing BITC concentration (lanes 4–6), a strong intensity decrease was noted (band I), while on the other hand, a clear intensity increase of a new band (green arrows) could be seen. Immunological detection showed certain differences with increasing concentration of BITC, indicating a change of the allergenic properties induced by ITC conjugation (Figure 2b). The blue coloration resulted from an antigen-antibody reaction, so that an increase in the color intensity could be equated with an increase in the immunological reaction. Conversely, a decrease in intensity of a band indicated an inhibition of the antibody response.

It is conceivable that the amino acid residues that covalently interact with ITC are sometimes located in an epitope. When amino acids in an epitope are conjugated with ITC or an ITC-protein bond is directly in the region or close to a linear epitope, the epitope can be blocked, inactivated, or destroyed (Figure 4c, loss of orange regions). The epitope is then no longer accessible for an antibody reaction [7], as reflected by a decrease in intensity of the blue coloration of the bands in Figure 2b (band I).

In addition to the shielding/masking of the epitopes by the covalent binding of ITC, the irreversible structural change of the protein molecule induced by ITC conjugation could cause the decrease in the intensity of blue staining, i.e., a decreased binding to antibodies.

A change in the structural properties of proteins as a result of the conjugation with ITC has been already observed in previous studies. Thus, a loosening of protein folding and a change in secondary and tertiary structure could be noted for an ITC conjugation to a whey protein isolate [6] and pure β-LG [3]. In addition, the results suggested that ITC could also play a role in the cleavage of disulfide bridges in β-LG [2,4,65,66]. Similar effects of ITC conjugation are conceivable for the protein α-LA.

As the conformation of the epitopes of α-LA is associated with its secondary or tertiary structure, which play an extraordinary role in the protein antigenicity, it is conceivable that a structural change induced by covalent ITC conjugation could presumably change some of the epitope structures and thus, affecting the allergenicity of the protein [60,63,67,68]. Based on these assumptions, a loosening of the protein molecule was mapped in Figure 4d (loss of orange regions), which may lead to the destruction or inactivation of conformational (discontinuous) epitopes under certain circumstances. A more significant change in the protein structure is shown in Figure 4e. Here, the fact that the ITC could cleave the disulfide bridges is pictorially illustrated. The result is a significant unfolding of the protein structure, corresponding to a certain extent of secondary structure transformation, but least strong transformation of the tertiary structure. These unfolding and denaturation processes may also inactivate and destroy conformational (discontinuous) and/or linear (sequential and continuous) epitopes (Figure 4d, further loss of orange regions), which could explain the reduction in intensity or disappearance of the bands in Figure 2b, lanes 4–6 (band I).

In addition to the destruction or inactivation of epitopes, it can be seen that as a result of the structural change, neo-formed epitopes occur or epitopes that were previously hidden inside (“buried”) the protein structure can become more accessible and exposed through unfolding and denaturation processes (Figure 4d,e, new orange regions), thereby increasing the immunological response. This effect of ITC conjugation on the antigenicity could explain the significant increase in intensity of the blue coloration of the band marked with green arrows in Figure 2b, lanes 5 and 6.

A purely hypothetical approach is shown in Figure 4f. Here, it is assumed that the protein is fully derivatized and completely fold over, after being exposed to an excessive BITC-modification. Furthermore, it is not known, whether epitopes can be completely destroyed or partially preserved in this state.

In summary, epitopes located on the surface of protein molecules can react with antibodies, while amino acids hidden inside cannot be recognized in the first moment. External influences and post-translational modifications such as the covalent binding of BITC as in this case, can cause the protein to denature and unfold, and consequently affecting protein structure with its physicochemical and biological properties. Irreversible structural changes of proteins resulting from covalent modifications can lead to antigenic epitopes either being blocked, destroyed, remodeled, or of even improved accessibility, thereby affecting the allergenic properties. On the one hand, the formation of neo-formed epitopes or the exposure of epitopes, initially buried inside the protein can lead to the formation of new, potentially immunogenic sequences or conformations. On the other hand, epitopes may be blocked or inactivated by ITC conjugation or conformational (discontinuous) epitopes may be destroyed by the structural change, resulting in a decreased antigen-antibody response.

In the following, the influence of ITC conjugation on enzymatic hydrolysis and on the antigenicity of tryptic peptides is discussed. Initially, the theoretically generated peptides upon enzymatic hydrolysis of α-LA with trypsin can be calculated along with the masses and positions of the peptides in the protein, using the PeptideMass tool available at www.expasy.org (accessed on 20 March 2021) (Table 2), The protein sequence of α-LA was taken from the UniProtKB database (http://www.uniprot.org/ (accessed on 20 March 2021); under the UniProt entry name LALBA_BOVIN, and file number P00711).

Comparison of protein-specific staining with fluorescamine and immunological staining using antibodies allows differentiation and assigning of antigenic and non-antigenic peptides. It should be emphasized that a specific antibody can bind to residual epitopes consisting of only a few residues of the initial antigen. For this purpose, the sequential epitope must consist of at least five linear arranged amino acids and can be up to a multiple number, when the linear arranged amino acids are coiled in the polypeptide [69]. It has already been shown that peptides consisting of 12–14 linear arranged amino acids with a molecular weight of approximately 1500 Da are responsible for a significant contribution to the allergenicity of the entire molecule [61,70]. Taking into account the relevant order of magnitude for an epitope character, the peptide sequences listed in Table 2 and the associated masses can be used to describe that are responsible for the antigen-antibody reaction, i.e., the appearance of the blue band in Figure 3b.

In Figure 4a, the tryptic cleavage sites are shown as green dashed lines, and the resulting peptides reflect the bands in Figure 3a, lane 1. Figure 3a showed a chromatogram followed by peptide-specific staining with fluorescamine. The high concordant band pattern of the two control samples (Figure 3a, lanes 1 and 2) indicated that the conditions of synthesis and re-conditioning had no effect on enzymatic hydrolysis. Only in lane 2 a slight but uniform reduction in the intensity of all bands was observed, which is probably due to the procedure, leading to minor losses, at all. In the chromatogram, a difference in the band pattern of the tryptic peptides with increasing degree of protein modification can be seen. From lanes 3–6, a loss of intensity as well as a decrease in the number of bands (red dashed boxes) was observed with increasing BITC concentration. It can be assumed that the formation of BITC adducts prevented or inhibited enzymatic hydrolysis with the protease trypsin. In Figure 4, the inhibition of enzymatic hydrolysis due to the conjugation is represented by red crosses and fewer green dashed lines. This trend increases from Figure 4b–f with increasing protein modification. This assumption can be underlined at hand of previous studies that have already investigated the influence of an AITC conjugation to β-LG with regard to digestibility [4]. It is well known that ITC can form stable thioureas with ε-amino groups of lysine side chains [71]. Based on the fact that trypsin preferentially cleaves peptide bonds after lysine and arginine, it seems to be obvious that the protein modification would result in a reduced tryptic digestibility [4,8,71].

Immunological staining revealed that of approximately 14 peptides separated, presumably three peptides were still immunologically-active, despite hydrolysis of the initial protein (Figure 3a,b, lane 1; yellow arrows). It is important to keep in mind that it cannot be ruled out that the chromatographic separation of the peptides was complete [52]. Overlapping of several peptides in one band is possible. Epitopes that are immunologically-active after enzymatic hydrolysis can be identified in Figure 4a by the fact that regions between the tryptic cleavage sites are highlighted in orange. When a tryptic cleavage site is located in the middle of an epitope, enzymatic hydrolysis at this site would destroy the epitope, being reflected by the reduced number of detected bands in Figure 3b.

In the immunological detection of the antigenic peptides, a significant decrease in the intensity of the blue coloration of the bands highlighted by a red dashed box was observed with increasing degree of protein modification (Figure 3b). Of presumably three immunologically-active peptides that still seem to provide an epitope, despite enzymatic hydrolysis of native α-LA, only one antigenic peptide was still detectable with increasing protein modification. The results showed that a certain degree of antigenicity was retained after enzymatic hydrolysis. It may either lead to an inhibition of enzymatic hydrolysis as a result of covalent binding, so that the immunologically-active peptide can no longer be formed, or the conjugation of ITC is close to or directly in the region of an epitope, which may have been blocked, inactivated, or destroyed. Inactivation of some epitopes by ITC conjugation is represented by red crosses and a loss of orange regions (e.g., Figure 4f).

Although some studies already investigated the influence of a protein modification with ITC on functional properties of the proteins, there is insufficient information on the influence on biological properties such as allergenicity. The current state of knowledge shows that the allergenic potential of foods can be reduced, unchanged, or even increased by various procedures in manufacturing and processing, such as mechanical, thermal, biochemical, or chemical treatments [46]. Initially, the recipe itself plays a pre-requisite role: ITC and proteins have to be present to a certain amount. As a result of food processing, the following structural changes of protein molecules are conceivable: Denaturation, unfolding, degradation, fragmentation, aggregation or the formation of oligomers, or neo-formed conformations. These changes in protein structure can also affect the epitope structures and thus, the allergenicity of the proteins [72,73,74]. The molecular basis for the change in allergenic properties is the modification of epitopes. Inactivation or destruction of epitopes, formation of new epitopes, or improved accessibility of previously hidden epitopes may occur [45,46,75]. Previous studies reported that the effect of heat treatment on proteins may lead to protein denaturation or aggregation. Consequently, the process-induced denaturation leads to the loss of the organized protein structure, which does not necessarily result in a reduced allergenicity. Rather, it is even conceivable that the formation of aggregates increases the allergenic potential, as illustrated in Figure 4g [61]. It is well known that aggregated proteins are considered to have high immunogenicity, because they can be readily taken up by antigen-presenting cells [76].

Furthermore, the influence of the food matrix during processing must be considered, because allergenic food proteins can interact chemically with other food components. Previous studies reported that new epitope structures can be formed, when proteins are heated in the presence of carbohydrates, polyphenols, or oxidized lipids [39,41,45,77]. Stanic-Vucinic et al. (2013) reported that chemical modification of β-LG is associated with structural and functional modification of proteins, which in turn may increase their allergenic potential by exposing hidden epitopes or creating new epitopes [78]. While the reaction of β-LG with lactose in milk showed increased allergenic activity [79], the allergenicity could be reduced by the covalent modification of ovalbumin [80] and the whey protein β-LG [7,81] with polyphenols in vitro.

In summary, chemical modifications of food proteins are often accompanied by protein unfolding and aggregation. Therefore, a loss of some structural elements and a change in the tertiary and secondary structure of the proteins can be expected [45].

In one study, the effect of heat exposure on the model whey protein α-LA was investigated by NMR. It was shown that the protein was partially unfolded, and the surface hydrophobicity of the protein changed. Residues previously buried in the hydrophobic core of the native protein thus reached the protein surface. Such structural changes have the potential to affect protein digestibility and allergenicity [45,82].

Apart from this, biochemical processes are known to potentially support the manufacturing of hypoallergenic foods, as hydrolysis of proteins showed reduced allergenicity [46].

Depending on the enzyme applied and the degree of hydrolysis, controversial observations are described in the literature. In case of incomplete hydrolysis of proteins, residual allergenicity could be attributed to the presence of native protein remaining or large fragments thereof. In the case of advanced hydrolysis, residual allergenic activity may be induced by short peptide fragments. Thus, short peptide fragments can account for a considerable amount of the allergenic activity of the entire protein. In native proteins, these peptide fragments are located in the hydrophobic core of the protein and are only exposed after protein denaturation or enzymatic hydrolysis. Thus, the remaining protein can still act as an allergen after hydrolysis [61].

## 4. Materials and Methods

### 4.1. Materials

1,4-Dioxane, 3,3′,5,5′-tetramethylbenzidine, acetonitrile, ammonia, polyethylene-sorbitan monolaurate (Tween20), sodium hydrogen carbonate, tris-HCl, tris(hydroxymethyl)-aminomethane, and dialysis membranes (regenerated cellulose, molecular weight cutoff < 3.5 kDa) were obtained from Carl Roth GmbH & Co. KG, Karlsruhe, Germany. Sodium chloride and tert-butanol were purchased from VWR International GmbH, Darmstadt, Germany. Bovine α-lactalbumin (α-LA) as model protein, benzyl isothiocyanate (98%), citric acid monohydrate, dioctyl sulfosuccinate sodium salt, dithiothreitol (DTT), fluorescamine, hydrogen peroxide, and trypsin from porcine pancreas were purchased from Sigma-Aldrich GmbH, Steinheim, Germany. The polyclonal primary antibodies were raised in rabbit against bovine α-LA with a concentration of 1g/L and obtained from antibodies-online GmbH, Aachen, Germany. A polyclonal goat anti-rabbit antibody, conjugated to HRP, was used as secondary antibody (Dako GmbH, Glostrup, Denmark). The concentration of the purified antibodies was 0.25 g/L. Ethanol, formic acid, hydrochloric acid, pyridine, urea, and HPTLC silica gel 60 were purchased from Merck KGaA, Darmstadt, Germany. The stationary phase materials were glass-backed and had a size of 10 × 20 cm. C_18_ solid phase extraction cartridges (1 mL, 100 mg) were purchased from Macherey-Nagel GmbH & Co. KG, Düren, Germany. All solvents were of HPLC grade, otherwise, ACS grade was used. Water was double distilled (ddH2O).

### 4.2. Methods

#### 4.2.1. Preparation of ITC Protein Conjugates

The protein solutions were prepared by dissolving α-LA in water (0.714 mM). Protein modification was conducted with four different BITC concentrations, ranging from 0 and 113 mM. BITC dissolved in 1,4-dioxane was added to the protein solutions and stirred for 20 h at 37 °C. After the incubation was finished, the reaction mixtures were dialyzed overnight and subsequently lyophilized for removing as much as residual BITC. For the lyophilization, all samples were frozen in liquid nitrogen to prevent cold denaturation and freeze-dried using a laboratory freeze dyer (Christ RVC 2−25 CDplus, Martin Christ Gefriertrocknungsanlagen GmbH, Osterode am Harz, Germany). The freeze-dried samples were dissolved in ACN/water (60/40; *v*/*v*), stored at −20 °C until analysis. For one of the control samples, the protein solution was treated in the same way as the modifications, except that BITC was not added.

#### 4.2.2. Tryptic Digestion of α-Lactalbumin

For the tryptic digestion, the freeze-dried ITC-modified and non-modified protein samples were dissolved in 50 µL 6 M aqueous urea. Subsequently, 100 mM DTT (in 100 mM sodium hydrogen carbonate) were added and incubated for 10 min at 60 °C to cleave any disulfide bridges. The samples were diluted with 425 µL 100 mM sodium hydrogen carbonate (in water). Tryptic digestion was started by adding trypsin solution (1 mg/mL in 0.1 mM HCl) to the protein solution at a ratio of 1:100. The incubation was performed for 16 h at 37 °C. After the incubation period, the reaction was quenched by adding 0.2% formic acid. For purification of the digested samples, a solid phase extraction was performed. Therefore, the chromatography material was conditioned with 60% ACN in water and equilibrated with 0.2% formic acid in water. After the application of the samples, rinsing was performed with 0.2% formic acid in water, followed by elution with 60% ACN. Finally, the samples were dried using gaseous nitrogen and re-dissolved in a defined volume of 0.2% formic acid for further analysis.

### 4.3. High-Performance Thin-Layer Chromatography-Immunostaining (HPTLC-IS)

#### 4.3.1. Protein Derivatives

Primarily, silica gel HPTLC plates were pre-washed with methanol and activated at 100 °C for 10 min. Sample application was done using an HPTLC autosampler (ATS4, CAMAG AG, Muttenz, Switzerland). A total sample volume of 10 µL was applied as 6 mm bands. Each sample was applied onto the plate in duplicate so that it could be cut in half after chromatographic separation, resulting in two identical plates (“twin plates”). The development of the HPTLC plates was performed in a saturated twin-trough chamber using a solvent system consisting of 2-butanol/pyridine/ammonia/water (39/15/10/36; *v*/*v*/*v*/*v*) adapted from Biller et al. [48]. After the solvent front reached a height of 80 mm, the plate was removed, and residual solvents were evaporated overnight.

#### 4.3.2. Tryptic Peptides

Analogously to the chromatographic analysis of the protein derivatives, the separation of the digested protein derivatives was performed. By using an HPTLC autosampler, samples were applied onto the plates and developed in a twin-trough chamber filled with the mobile phase consisting of 2-butanol/pyridine/ammonia/water (39/34/10/26; *v*/*v*/*v*/*v*) [52]. Finally, residual solvents were evaporated from the plates.

#### 4.3.3. Protein-/Peptide-Specific Staining

After the residual solvents were evaporated overnight, proteins and peptides were stained with fluorescamine. Visualization of the fluorescent proteins/peptides was performed using a photodocumentation system (TLC-Visualizer, CAMAG AG, Muttenz, Switzerland) under ultraviolet light (UV 366 nm). The proteins/peptides appeared as fluorescent bands on a dark background. These plates were used as twin plates to compare with the immunostained plates.

#### 4.3.4. High-Performance Thin-Layer Chromatography-Immunostaining (HPTLC-IS)

Specific detection of proteins and peptides were performed using antibodies according to the procedure described by Morschheuser et al. [52]. Following the evaporation of the mobile phase overnight, one half of the HPTLC plate was transferred to a small vessel.

First, the HPTLC plate was incubated two times for 15 min with a buffer containing Tween20 as a blocking reagent. This prevents the primary antibody from binding non-specifically to the plate surface. For preparing the blocking solution, 0.9% sodium chloride, 0.6% tris(hydroxymethyl)aminomethane, and 0.5% Tween20 was used. Subsequently, the blocking buffer was removed and replaced by the primary antibody solution (1:20,000 for intact proteins and 1:12,000 for peptides, both in blocking buffer). The incubation with primary antibodies lasted for 2 h and was followed by three washing steps with the blocking solution. Coating with the secondary antibody was performed for 1 h (diluted 1:2000 in blocking solution). The secondary antibody exhibited specificity against the primary antibody and was HRP-conjugated. After the plates were washed three times with the blocking buffer, pH was lowered by incubation for 1 min with an acidic solution containing 0.06% Tris-HCl (pH 6.0). The staining solution consists of the following substances: 0.06% 3,3′,5,5′-tetramethylbenzidine, 0.2% dioctyl sulfosuccinate sodium salt, 0.7% citric acid monohydrate, 1.8% sodium hydrogen phosphate dihydrate, 25% ethanol. Just before staining, 0.15% hydrogen peroxide was added. The TLC plate was incubated in the staining solution for 10 min until blue bands appeared. Upon oxidation, TMB forms a water-soluble blue reaction product that can be measured spectrophotometrically at 650 nm. All incubations were executed at room temperature and on an orbital shaker at 70 rpm. Finally, the analytes were detected at white light and documented using a photodocumentation system (TLC visualizer, CAMAG AG, Muttenz, Switzerland).

### 4.4. Statistical Analysis

Validation of the HPTLC-IS methodology was performed with regard to following parameters: linearity, precision, accuracy, limit of detection (LOD), and limit of quantification (LOQ). For this purpose, native α-LA was used as the standard compound and treated like the modification except for the addition of BITC for excluding any effect of treatment and reconditioning. Subsequently, a calibration series was prepared from the treated non-modified α-LA stock solution. The standard solutions were separated on silica gel and then detected by immunological reaction with antibodies.

Following the documentation of the plates, densitometric evaluation was performed at 650 nm using a TLC Scanner 4 (CAMAG AG, Muttenz, Switzerland). Densitograms recorded with the TLC scanner can be used for quantitative analysis. Linearity was validated using a 15-point calibration curve (n = 3) and was verified by means of Mandel’s fitting test. Homogeneity of variance was assigned regarding all values obtained [83,84].

Limit of detection (LOD) and limit of quantification (LOQ) were determined using the calibration method DIN 32645:2008-11 (n = 3) via the calibration curve (concentrations between 0.2–3 µg). Accuracy and precision were determined by analyzing the closeness of individual densitometric measurements of the band (same concentration of α-LA, c = 1.8 µg, n = 12).

## 5. Conclusions

In summary, immunological staining of native untreated and BITC-modified α-LA could be successfully performed directly on the plate after their separation using HPTLC. As the HPTLC-IS procedure does not destroy the tertiary and secondary structure, it was possible to estimate the antigenic properties of the modified and non-modified proteins. In the present work, even highly degraded samples such as denatured or proteolytically digested proteins could be detected. It is obvious that due to the chemical modification of α-LA with BITC, structural changes of the protein molecule potentially influence the allergenic properties. Consequently, the chemical modifications could have destroyed or inactivated epitopes, but at the same time buried or newly formed epitopes can be exposed, so that the net effect was an increase in allergenicity. It can be concluded that despite protein modification, (some of the) epitopes remained immunologically-active, at least.

Regarding the enzymatic hydrolysis with trypsin of the BITC-modified proteins, it can be concluded that the conjugation of BITC with free amino groups of lysine protein side chains probably sterically hindered the cleavage sites for the enzyme and consequently led to a reduced digestibility and altered peptide patterns. While for the native and untreated α-LA three of the 14 separated peptides could be detected immunologically, the BITC-treated samples showed only one antigenic peptide. However, it is possible that not every band can be assigned to a single peptide, but several peptides may overlap in one band.

For this purpose, coupling with mass spectrometry of HPTLC-IS could provide excellent features for the advanced studies of antigens. It has to be considered that the blocking reagent Tween20 can interfere with the signals in mass spectrometry. Therefore, the immunostaining procedure has to be optimized with regard to blocking agents. Other major advantages of HPTLC-IS are its ease of use, low cost compared to alternative methods such as gel electrophoretic separation on membranes and staining afterwards, and the reduced use of toxic substances such as acrylamide in SDS-PAGE. In addition, antibody-based detection is characterized by high affinity and specificity to target proteins. Due to the high variability of the HPTLC analysis, e.g., with regard to the composition of the mobile phases, the conditions of the HPLC-IS can be individually adapted to the analytical problem at hand. Furthermore, the methodology profits from a two-dimensional development, which can increase the chromatographic resolution. Here, the sample of interest is developed in two directions with different composition of the mobile phase. Initial experiments of two-dimensional HPTLC-IS have already been performed, which seem promising and should therefore be further optimized in future research.

It is of particular importance to protect sensitized individuals from an allergenic reaction. For this purpose, a method that ensures the identification of allergenic proteins is mandatory. As only an estimation of the antigenicity of the native and the processed protein is obtained with the method described here, future studies need to evaluate and compare the immunogenicity and allergenicity with other analytical methods such as enzyme-linked immunosorbent assays, radio-immunoprecipitate assays, electrochemiluminescence, bead-based assays, surface plasmon resonance or bio-layer interferometry. In addition, specific assays for the determination of protein antigenicity are available [85,86]. In addition, there is no clear tendency to what extent the various food processing influences the allergenic properties [45], so furthermore exhaustive studies are necessary. For example, processing techniques may reduce or increase the bovine allergen in cow’s milk or leave it unchanged at all [46]. The evaluation of such non-directed posttranslational modifications that can occur frequently in foods remains challenging. In addition to in vitro studies, it is also necessary to verify, whether the findings obtained can be observed in vivo.

## Figures and Tables

**Table 1:** Statistical parameters of HPTLC-IS using anti-bovine α-LA antibodies.

Synonym	Value	RSD
Limit of detection	0.040 µg	0.74%
Limit of quantification	0.177 µg	3.27%
Accuracy	99.93%	4.27%
Precision	8.55%	-
Coefficient of determination	0.956	1.52%

**Table 2:** Masses, positions in the protein and sequences of the calculated peptides after tryptic hydrolysis using the PeptideMass tool available at www.expasy.org (accessed on 20 March 2021).

Mass	Position	Peptide Sequence
618.35	20–24	EQLTK
653.31	25–29	CEVFR
389.24	30–32	ELK
375.22	33–35	DLK
4654.15	36–77	GYGGVSLPEWVCTTFHTSGYDTQAIVQNNDSTEYGLFQINNK
549.29	78–81	IWCK
1889.78	82–98	DDQNPHSSNICNISCDK
1642.73	99–112	FLDDDLTDDIMCVK
147.11	113–113	K
488.31	114–117	ILDK
1220.65	118–127	VGINYWLAHK
650.32	128–133	ALCSEK
1034.50	134–141	LDQWLCEK
132.10	142–142	L

## Data Availability

The data presented in this study are available on request from the corresponding author.

## Sample Availability

Samples of the compounds are available from the authors.
